# Acute Postprandial Glycemic and Antioxidant Responses to Maqui Berry Gummies in Adults with Overweight/Obesity: A Small Within-Subject Pilot Study

**DOI:** 10.3390/ijms27209133

**Published:** 2026-10-15

**Authors:** Paula Acevedo-Hernández, Viviana Valero-Jara, Carlos Jara-Gutiérrez, Claudia Ibacache-Quiroga, Gonzalo Jorquera, Samanta Thomas-Valdés

**Affiliations:** 1 Centro Interdisciplinario de Neurociencia de Valparaíso (CINV), Facultad de Ciencias, Universidad de Valparaíso, Valparaíso 2362905, Chile; paula.acevedo@uv.cl; 2 PhD Program in Health Sciences and Engineering, Facultad de Medicina, Universidad de Valparaíso, Valparaíso 2362905, Chile; viviana.valero@uv.cl; 3 Centro Interdisciplinario de Investigación Biomédica e Ingeniería para la Salud-MEDING, Escuela de Kinesiología, Facultad de Medicina, Universidad de Valparaíso, Valparaíso 2362905, Chile; carlos.jara@uv.cl; 4 Centro de Micro-Bioinnovación, Facultad de Farmacia, Escuela de Nutrición y Dietética, Universidad de Valparaíso, Valparaíso 2361814, Chile; claudia.ibacache@uv.cl; 5 Instituto de Fisiología, Facultad de Ciencias, Universidad de Valparaíso, Valparaíso 2361814, Chile; 6 Instituto de Nutrición y Tecnología de los Alimentos (INTA), Universidad de Chile, Santiago 7810430, Chile

**Keywords:** *Aristotelia chilensis*, maqui berry, microencapsulation, postprandial glycemia, GLP-1, insulin, antioxidant biomarkers, gummy candy

## Abstract

Maqui berry is rich in anthocyanins and has attracted interest as a functional food ingredient with potential metabolic and antioxidant effects. However, little is known about its acute postprandial effects in gummy formulations or whether microencapsulation modifies sensory or biological responses. Ten adults with overweight or obesity completed three acute conditions in a fixed sequence: white bread alone (Control), white bread with unencapsulated maqui berry gummies (UMBG), and white bread with microencapsulated maqui berry gummies (MMBG). Glucose was assessed over 120 min; insulin, glucagon-like peptide-1 (GLP-1), superoxide dismutase (SOD), catalase, and total reactive antioxidant power (TRAP) were assessed at baseline and 120 min. Sensory acceptability was evaluated separately in 66 panelists. Both formulations showed generally acceptable sensory profiles. In the reported analyses, co-consumption of gummies was associated with lower glucose concentrations (*p* < 0.05), and total glucose AUC was lower under MMBG than under Control (*p* < 0.0207). Insulin at 120 min was higher under both gummy conditions (*p* < 0.01), and GLP-1 was higher under MMBG (*p* < 0.05); however, changes in GLP-1 from baseline did not differ significantly among conditions (*p* > 0.05). Direct comparisons of baseline-adjusted analyses in antioxidant biomarkers established a differential response among gummies formulations, with UMBG exhibiting better TRAP results with decrement of SOD activity when adjusted for the corresponding baseline value. These preliminary observations warrant further investigation. The small sample, fixed intervention sequence, and absence of a matched placebo gummy limit causal attribution. The reported findings do not establish antioxidant protection or superiority of microencapsulation and require confirmation in an adequately powered randomized trial.

## 1. Introduction

Postprandial metabolic responses are increasingly recognized as relevant indicators of metabolic health, particularly in individuals with overweight or obesity [1,2]. After ingestion of carbohydrate-rich foods, transient elevations in blood glucose can promote oxidative stress, reduce circulating antioxidant capacity, and contribute to impaired glucose homeostasis when repeated over time [3,4]. Therefore, dietary strategies capable of attenuating acute postprandial glycemic excursions while supporting antioxidant defenses are of interest for the development of functional food approaches.

Polyphenol-rich berries have received particular attention in this context. Anthocyanins and related phenolic compounds can influence postprandial glucose metabolism through several complementary mechanisms, including inhibition of carbohydrate-digesting enzymes, modulation of intestinal glucose transport, effects on incretin secretion, and antioxidant activity [5,6]. Previous human studies using berries or anthocyanin-rich extracts have reported favorable effects on postprandial glycemia, insulin responses, and oxidative-stress-related biomarkers [3,7,8,9], although the magnitude of these effects depends on the food matrix, dose, population, and study design [10].

Maqui berry (*Aristotelia chilensis*), a native Chilean fruit, is characterized by high content of anthocyanins, particularly delphinidin and cyanidin derivatives [11,12]. These compounds have been associated with antioxidant and glucose-modulating properties, making maqui berry a promising ingredient for functional food development [13,14]. In the present study, characterization of the maqui berry powder confirmed the presence of major delphinidin and cyanidin derivatives, supporting its use as a polyphenol-rich ingredient (Supplementary Table S1).

Despite this potential, incorporating anthocyanin-rich ingredients into food products presents important technological and sensory challenges. Anthocyanins are sensitive to processing conditions, pH, oxygen, temperature, and storage, which may reduce their stability and biological activity [15,16]. Polyphenols can also contribute bitterness, astringency, and intense color or aroma, potentially limiting consumer acceptability [17]. Microencapsulation, particularly spray drying with carriers such as maltodextrin, is used to protect sensitive compounds and modify their handling, stability, release, and sensory characteristics [18,19]. These potential advantages depend on the bioactive ingredient, encapsulation procedure, and food matrix and do not necessarily translate into greater acceptability or biological efficacy [19].

Gummy candies represent a convenient food matrix for delivering bioactive ingredients [20]. Because conventional gummies are often sugar-containing products, their use as functional food vehicles requires careful evaluation when the intended outcome concerns glycemic responses [21]. Previous formulation studies have examined sensory attributes [22,23], phytochemical bioaccessibility and stability [19,24,25], and alternatives to conventional gummy ingredients [26]. Evaluation in human participants is needed to connect formulation characteristics with postprandial responses. In this context, developing a maqui berry gummy should consider sensory acceptability and polyphenol delivery together with the acute metabolic response to co-consumption with a carbohydrate-rich meal.

Evidence on acute postprandial responses to maqui berry incorporated into gummy formulations remains limited. This exploratory within-subject pilot study aimed to develop and characterize maqui berry gummies, compare the sensory acceptability of microencapsulated and unencapsulated formulations, and examine postprandial glycemic, hormonal, and antioxidant-response biomarkers after co-consumption with a standardized white bread challenge in adults with overweight or obesity. The formulation comparison examined whether microencapsulation was associated with differences in sensory acceptance or acute response patterns. The fixed-sequence design and small sample require an exploratory interpretation of these comparisons.

## 2. Results

### 2.1. Maqui Berry Powder Characterization and Gummy Formulation

HPLC-UV/Vis analysis of the methanolic extract of maqui berry powder was performed to characterize its anthocyanin profile. Five major anthocyanin-related peaks were detected and tentatively assigned as delphinidin and cyanidin derivatives based on their retention times and UV-visible spectra (Figure S1 and Table S1). Delphinidin derivatives accounted for the largest proportion of the chromatographic area, supporting the use of this powder as an anthocyanin-rich ingredient for gummy formulation (Table S1). In parallel, the total polyphenolic content of the final gummy products was determined using the Folin–Ciocalteu method and expressed as quercetin equivalents (QE). The total polyphenolic content was 28.87 mg QE/g of gummy, corresponding to 231 mg QE per gummy. Therefore, the intervention serving of three gummies provided 693 mg QE per participant.

### 2.2. Sensory Acceptability of Maqui Berry Gummies

Both gummy formulations received neutral to positive sensory acceptability ratings across the evaluated attributes, with median scores at or above the midpoint of the five-point hedonic scale (Figure 1 and Table 1). No significant differences between UMBG and MMBG were observed for appearance, color, bitterness, astringency, sweetness, softness, smoothness, and taste (Table 1). In the current analysis, UMBG received higher acceptability scores than MMBG for smell (Table 1). A significant difference was detected only for smell; the remaining attributes did not show statistically significant differences. These non-significant comparisons do not establish equivalence between formulations.

### 2.3. Participant Characteristics and Estimated Background Dietary Intake

Ten adults with overweight or obesity completed the acute within-subject intervention, including seven women and three men. Participants had a mean age of 38.9 ± 11.9 years, a body weight of 84.6 ± 12.1 kg, a waist circumference of 101.2 ± 6.9 cm, a BMI of 31.1 ± 3.6 kg/m^2^, and a triceps skinfold thickness of 35.3 ± 9.7 mm.

Estimated background dietary intake of fiber, vitamin C, carotenoids, vitamin E, and polyphenols did not differ significantly across the control, UMBG, and MMBG intervention periods (Table S2). Because these estimates were derived from a food frequency questionnaire, they were used to characterize the participants’ habitual dietary context rather than dietary intake on the specific intervention days.

### 2.4. Antioxidant Response Biomarkers

Antioxidant-response biomarkers were assessed at baseline and 120 min under each intervention condition (Table 2). The reported within-Control comparisons yielded p=1.00 for CAT, p=0.05 for SOD, and p=0.03 for TRAP. The corresponding within-condition results for UMBG and MMBG are presented in Table 2.

The reported between-condition comparisons were analyzed using a linear mixed-effects models considering each intervention (Control, UMBG, and MMBG) as a fixed effect and the corresponding baseline value measured at each intervention visit as a covariate (Table 3). We observed a significant effect of the intervention on SOD at 120 min after adjustment for baseline SOD (F(2,23) = 5.94, *p* = 0.0083). The baseline SOD value was not significantly associated with SOD at 120 min (F(1,23) = 1.77, *p* = 0.197). Holm-adjusted post hoc comparisons showed that SOD was higher in Control versus UMBG (mean difference = 0.47, SE = 0.14, *p* = 0.0074), whereas no significant differences were observed between Control and MMBG (*p* = 0.1227) or between UMBG and MMBG (*p* = 0.1227). Estimated marginal means were 2.06, 1.59, and 1.80 for Control, UMBG, and MMBG, respectively. For CAT, the linear mixed-effect model showed no significant effect of the intervention at 120 min after adjustment for baseline CAT values (*F*(2,23) = 0.19, *p* = 0.829). The baseline CAT value was significantly associated with CAT at 120 min, (*F*(1,23) = 15.10, *p* = 0.00075). Holm-adjusted post hoc comparisons showed no significant differences between Control and UMBG (mean difference = 0.05, SE = 0.07, *p* = 1.000), Control and MMBG (mean difference = 0.03, SE = 0.07, *p* = 1.000), or UMBG and MMBG (mean difference = −0.01, SE = 0.07, *p* = 1.000). For TRAP, the linear mixed-effects model showed a significant effect of the intervention on TRAP at 120 min after adjustment for baseline TRAP (*F*(2,17.84) = 5.11, *p* = 0.0176). The baseline TRAP value was not significantly associated with TRAP at 120 min (*F*(1,22.49) = 4.07, *p* = 0.0556). Holm-adjusted post hoc comparisons showed that TRAP was higher in UMBG compared with Control (mean difference = 0.59, SE = 0.19, *p* = 0.0202), while in UMBG presented higher values than in MMBG (mean difference = 0.78, SE = 0.28, *p* = 0.0207), whereas no significant difference was observed between Control and MMBG (mean difference = 0.19, SE = 0.19, *p* = 0.3458). Estimated marginal means were 2.33, 2.92, and 2.14 for Control, UMBG, and MMBG, respectively. After adjustment for visit-specific baseline values, SOD activity at 120 min was lower under UMBG than under Control, whereas TRAP was higher under UMBG than under Control and MMBG. No significant condition effect was observed for CAT. These exploratory findings do not establish antioxidant protection.

**Table 3:** Baseline-adjusted antioxidant biomarker values at 120 min across intervention conditions.

	Marginal Means ± Standard Error (SE)			
	Control	UMBG	MMBG	*p*
CAT (U/mg protein)	1.32 ± 0.05	1.27 ± 0.05	1.29 ± 0.05	0.8289
SOD (U/mg protein)	2.06 ± 0.10	1.59 ± 0.08	1.80 ± 0.08	0.0083
TRAP (TEAC μM)	2.33 ± 0.13	2.92 ± 0.17	2.14 ± 0.17	0.0176

Data are presented as estimated marginal means ± standard error (SE) adjusted for the corresponding baseline value. SOD, CAT, and TRAP at 120 min were compared across the Control, UMBG, and MMBG conditions using linear mixed-effects models, with the intervention condition as a fixed effect, the corresponding baseline value as a covariate, and the participant as a random intercept. Control, 150 g of white bread consumed alone; UMBG, unencapsulated maqui berry gummy co-consumed with 150 g of white bread; MMBG, microencapsulated maqui berry gummy co-consumed with 150 g of white bread; CAT, catalase; SOD, superoxide dismutase; TRAP, total reactive antioxidant power. *p* < 0.05 was considered statistically significant. *N* = 10 participants.

### 2.5. Postprandial Glucose, Insulin, and GLP-1 Responses

Postprandial glucose concentrations increased after the white bread challenge in all three conditions (Figure 2a). The reported analysis showed significant main effects of time, F(2.885,69.24)=32.42, p<0.0001, and condition, F(2,24)=10.72, p=0.0123, as well as a significant time × condition interaction, F(10,120)=2.54, p=0.0082. Post hoc comparisons were performed to identify differences between conditions at individual postprandial time points. Compared with the Control condition, lower glucose concentrations were observed after co-consumption with UMBG and/or MMBG at selected postprandial time points (Figure 2a). Total glucose AUC differed significantly among the three conditions ($H\left(2\right)=7.432$, p=0.0243). Post hoc comparisons with multiplicity-adjusted p values showed that total glucose AUC was significantly lower after MMBG than after Control ($p_{adjusted}=0.0207$), whereas no significant differences were observed between Control and UMBG ($p_{adjusted}=0.2890$) or between UMBG and MMBG ($p_{adjusted}=0.8959$) (Figure 2b).

Insulin concentrations were assessed at baseline and 120 min after each intervention condition (Figure 3a–c). Fasting insulin concentrations did not differ significantly among the Control, UMBG, and MMBG conditions ($H\left(2\right)=2.550$, p=0.2795; Figure 3a). At 120 min, insulin concentrations differed significantly among conditions ($H\left(2\right)=16.320$, p=0.0003; Figure 3b). Post hoc comparisons with adjusted p values showed that insulin concentrations were significantly higher after UMBG than after Control ($p_{adjusted}=0.0010$) and after MMBG than after Control ($p_{adjusted}=0.0024$); no significant difference was observed between UMBG and MMBG ($p_{adjusted}>0.9999$). Changes in insulin concentrations from baseline to 120 min (Δ insulin) also differed significantly among the three intervention conditions (F(2,26)=13.32, p=0.0001; Figure 3c). Post hoc comparisons showed significantly different changes between Control and UMBG ($p_{adjusted}<0.0001$) and between Control and MMBG ($p_{adjusted}=0.0050$), whereas the difference between UMBG and MMBG was not statistically significant ($p_{adjusted}=0.3487$).

GLP-1 concentrations were assessed at baseline and 120 min after each intervention condition (Figure 3d–f). At baseline, GLP-1 concentrations did not differ significantly among the Control, UMBG, and MMBG conditions (F(2,17)=2.526, p=0.1096; Figure 3d). At 120 min, the overall comparison among conditions was significant ($H\left(2\right)=6.280$, p=0.0366; Figure 3e). Post hoc comparisons with adjusted p values showed significantly higher GLP-1 concentrations after MMBG than after Control ($p_{adjusted}=0.0383$), whereas no significant differences were observed between Control and UMBG ($p_{adjusted}=0.6121$) or between UMBG and MMBG ($p_{adjusted}=0.5743$). Changes in GLP-1 concentrations from baseline to 120 min did not differ significantly among the three intervention conditions (Figure 3f). Thus, although MMBG resulted in higher absolute GLP-1 concentrations at 120 min than the Control condition, the change from baseline analysis did not demonstrate a significant differential GLP-1 response among conditions.

### 2.6. Exploratory Correlation Analyses

Exploratory Spearman correlations were examined separately under UMBG and MMBG between estimated background dietary intake and metabolic or antioxidant variables (Table S3). Under UMBG, polyphenol intake was positively correlated with SOD (ρ=0.826, p=0.01), and vitamin C intake was positively correlated with glucose AUC (ρ=0.733, p=0.03). Under MMBG, the reported association between polyphenol intake and SOD was ρ=0.639 with p=0.05. These *p* values were unadjusted exploratory values. The small sample and number of correlations examined require cautious, hypothesis-generating interpretation and do not establish causality.

## 3. Discussion

In this exploratory within-subject pilot study, we examined acute postprandial responses to a white bread challenge with and without maqui berry gummies in adults with overweight or obesity. Both formulations received generally acceptable sensory ratings. The reported metabolic analyses identified lower glucose concentrations at selected time points and a lower total glucose AUC under MMBG than under Control. However, the direct MMBG versus UMBG comparison did not establish a statistically significant AUC difference. Direct comparisons of changes in antioxidant biomarkers established a differential response among gummy formulations, with UMBG exhibiting better TRAP results with decrement of SOD activity when adjusted for the corresponding baseline value. Hormonal observations require cautious interpretation because sampling was limited to baseline and 120 min. These findings describe preliminary response patterns; the small sample, fixed sequence, and absence of a matched placebo gummy limit causal attribution and do not establish a specific benefit of microencapsulation or antioxidant protection.

The maqui berry powder showed a profile dominated by delphinidin and cyanidin derivatives, consistent with previous descriptions of *Aristotelia chilensis* as an anthocyanin-rich fruit [11,12]. Spray-drying microencapsulation with maltodextrin has been used to improve the stability of sensitive phytochemicals during processing, storage, and gastrointestinal exposure [19,25]. Such technological advantages do not necessarily translate into greater sensory acceptance. In the present study, both formulations were generally acceptable, but UMBG received higher smell acceptability scores, and the remaining attributes did not show statistically significant differences. Processing conditions and carrier materials may influence sensory characteristics [18,27], although this study did not identify the cause of the smell difference. Previous work has also shown formulation-dependent acceptance, including lower acceptance of microencapsulated resveratrol gummies than their unencapsulated counterparts [22] and changes in sensory properties with plant-derived ingredients [23]. Microencapsulation should therefore be evaluated as a formulation variable without assuming a sensory advantage in a particular food matrix.

The reported glucose findings provide an exploratory signal for further investigation. Co-consumption of maqui berry gummies was associated with lower glucose concentrations at selected time points, and total glucose AUC was lower under MMBG than under Control. This comparison does not demonstrate the superiority of microencapsulation, because the direct comparison between MMBG and UMBG was not statistically significant. That non-significant comparison also does not establish equivalence. Stability, absorption, and circulating anthocyanin metabolites were not measured, so the observed response cannot be attributed to improved bioavailability from microencapsulation. Previous human studies provide biological context for the glucose-modulating effects of anthocyanin-rich foods: acute blueberry intake attenuated postprandial responses in adults with metabolic syndrome [7], and fresh blueberries co-consumed with white bread reduced glucose AUC in sedentary adults [28]. Apple polyphenol-rich beverages reduced early postprandial glucose concentrations in a dose-dependent manner [29], and a delphinidin-rich maqui extract reduced glycemia during oral glucose tolerance testing in people with prediabetes [30]. Dose–response blueberry interventions have also examined postprandial glucose and insulin responses [31], while other blueberry trials in healthy participants did not detect significant changes in glucose metabolism [32]. These studies illustrate variation across populations, doses, matrices, and protocols; they do not establish the mechanism or efficacy of the present gummies. Our comparisons with the Control remain limited by the fixed sequence and lack of a matched placebo gummy and do not demonstrate a definitive improvement in glycemic control.

The insulin and GLP-1 observations require cautious interpretation because measurements at baseline and 120 min cannot characterize complete postprandial trajectories or a meaningful full-response AUC. The reported fasting comparisons did not identify statistically significant differences. At 120 min, insulin was higher under both gummy conditions and GLP-1 was higher under MMBG than under Control. However, the change from baseline GLP-1 comparison did not demonstrate a significant differential response, so the higher absolute concentration at 120 min does not establish stimulation of GLP-1 by MMBG. Polyphenols may influence enteroendocrine signaling through gastrointestinal bitter taste receptors [5], and acute studies of catechins and chlorogenic acids have examined incretin responses [33]. Other polyphenol-rich interventions have reported varying glucose and hormonal responses [29,31]. This literature provides possible mechanisms for future investigation but does not demonstrate a mechanism in this study. Limited sampling, small sample size, fixed order, and the absence of a matched placebo gummy prevent causal or mechanistic conclusions from the present hormonal observations.

Antioxidant response findings require interpretation based on direct between-condition comparisons rather than separate within-condition significance patterns. In the analyses currently reported, changes in CAT from baseline to 120 min did not show differences; however, reduction of SOD and increment of TRAP results were significantly different for the UMBG formulation compared to the Control condition when adjusted for the corresponding baseline value. The UMBG values for the TRAP assay would be associated with more capacity of the compounds in this specific antioxidant assay than microencapsulated gummy. So, the reported findings therefore do not establish antioxidant protection or prevention of an oxidative response by either formulation. Baseline differences across visits require explicit evaluation, and the baseline-adjusted analyses do not substitute for baseline-adjusted analyses. Previous studies of anthocyanin-rich bread and yogurt have examined acute antioxidant capacity responses [3,34], whereas longer interventions with anthocyanin-rich or açaí and juçara juices have reported changes in antioxidant measures over several weeks [35,36]. Glucose load studies also provide context for acute changes in circulating antioxidant defenses [37,38]. These findings concern different exposures, populations, and sampling schedules and do not establish an effect in the present gummies. Any interpretation of baseline-adjusted results must also retain the limitation that intervention condition and study period cannot be separated in this fixed sequence.

The exploratory correlation analyses identified associations between estimated dietary polyphenol intake and selected metabolic and antioxidant variables, including the reported association between polyphenol intake and SOD activity under UMBG. These observations are biologically plausible, as dietary anthocyanins and other polyphenols have been associated with modulation of antioxidant defenses [39]. However, these correlations should be interpreted with particular caution. Dietary exposure was estimated using a food frequency questionnaire, the number of participants contributing to individual analyses was small, and multiple correlations were explored without adjustment for multiplicity. Consequently, these associations cannot establish a relationship between habitual polyphenol intake and the acute effects of the gummy interventions and should be regarded as hypothesis-generating observations for future studies. Larger trials incorporating objective measures of polyphenol exposure, such as circulating or urinary polyphenol metabolites, would be required to determine whether baseline dietary polyphenol status modifies the acute metabolic response to maqui berry supplementation.

Several strengths and limitations should be considered. The within-subject design reduces the contribution of interindividual variability to comparisons, but the small sample (*n* = 10) limits precision, generalizability, and the ability to detect differences, particularly for additional exploratory outcomes and correlations. No formal prospective efficacy-based sample size calculation was performed. All participants completed Control, UMBG, and MMBG in the same sequence, so condition was completely confounded with period. The observed differences cannot be attributed to intervention effects independently of period/order effects, and the direction and magnitude of any resulting bias are unknown. Standardized pre-test conditions and a one-week washout do not eliminate this limitation. The absence of a matched placebo gummy and lack of participant blinding also prevent effects specifically attributable to maqui-derived compounds from being separated fully from matrix or other intervention-related effects. Numerical baseline differences in antioxidant biomarkers require direct comparison and baseline adjustment; even such adjustment cannot resolve the fixed-order confounding. Insulin and GLP-1 sampling at baseline and 120 min prevents characterization of their complete trajectories. Questionnaire-derived diet estimates describe the preceding week rather than exact intake on the intervention days. Circulating anthocyanins or metabolites were not measured, so differences in bioavailability between formulations cannot be established; this is relevant to the extensive metabolism and biotransformation of anthocyanins [40]. The acute intervention cannot establish effects of habitual consumption or long-term health benefits. Future adequately powered trials should randomize and counterbalance sequences, include a matched placebo gummy, and obtain more extensive hormone sampling and relevant measures of anthocyanin exposure. Longer supplementation studies are needed to address sustained effects, and direct formulation comparisons are needed to assess any advantage of microencapsulation.

## 4. Materials and Methods

### 4.1. Chemicals and Reagents

Folin–Ciocalteu phenol reagent, ethylenediaminetetraacetic acid (EDTA), hydrogen peroxide, methanol, acetonitrile, formic acid, sodium carbonate, sodium chloride, sodium dihydrogen phosphate, and disodium hydrogen phosphate were obtained from Merck (Darmstadt, Germany). Ethanol, methanol, quercetin, maltodextrin, 2,2′-azobis(2-amidinopropane) dihydrochloride (AAPH), and 2,2′-azino-bis(3-ethylbenzothiazoline-6-sulfonic acid) diammonium salt (ABTS) were purchased from Sigma-Aldrich (St. Louis, MO, USA). Jelly berry flavoring was obtained from Vivo^®^-Carozzi (Santiago, Chile), and lyophilized maqui berry (*Aristotelia chilensis*) powder was obtained from Nanuva (Los Ángeles, Chile).

### 4.2. Characterization of Maqui Berry Powder and Gummy Formulations

The anthocyanin profile of lyophilized maqui berry (*Aristotelia chilensis*) powder was characterized through high-performance liquid chromatography with UV-visible detection (HPLC-UV/Vis) (Figure S1). For this purpose, lyophilized maqui berry was extracted three times with methanol: formic acid (99:1 *v*/*v*) in a 1:3 *v*/*v* homogenate: solvent ratio in the dark under sonication (15 min each). The combined extracts were taken to dryness under reduced pressure at 40 °C in a rotary evaporator and freeze-dried. Chromatographic analysis was performed using Jasco (Jasco Corporation, Tokyo, Japan) equipped with an LC-20AT pump, a UV-975 UV/Vis detector, and a CO-2067 Plus column oven maintained at 25 °C. Separation was achieved using a MultoHigh 100 RP18-5 column (250 × 4.6 mm, 5 μm; CS-Chromatographie Service GmbHLangerwehe, Germany). The chromatographic method was adapted from [41]. Mobile phase A consisted of water/acetonitrile/formic acid (87:3:10, *v*/*v*/*v),* and mobile phase B consisted of water/acetonitrile/formic acid (40:50:10, *v*/*v*/*v*). The flow rate was 1.0 mL/min, and the injection volume was 20 μL. The gradient was programmed from 94% to 70% A over 15 min, from 70% to 50% A over the following 15 min, and from 50% to 40% A over 5 min. Solvent A was then returned from 40% to 94% over 6 min, followed by 9 min of equilibration at 94% A. Chromatograms were monitored at 280, 320, and 520 nm. Major anthocyanin-related peaks were tentatively assigned as delphinidin or cyanidin derivatives based on retention characteristics and UV-visible spectral profiles and compared with the reference standards cyanidin-3-glucoside and del-phinidin-3-glucoside (Sigma-Aldrich).

The total polyphenolic content was determined in the mixture using the Folin–Ciocalteu colorimetric method, with modifications based on Ruiz et al. [42]. Briefly, 1 mL of the corresponding sample was used for the analysis. The sample was mixed with Folin–Ciocalteu reagent and sodium carbonate solution, according to the experimental protocol, and incubated under controlled conditions. Absorbance was subsequently measured at 765 nm using a spectrophotometer. Quercetin was used as the calibration standard, and the total polyphenolic content was expressed as mg quercetin equivalents (QE) per gram of gummy (mg QE/g) and per gummy (mg QE/gummy).

### 4.3. Microencapsulated Maqui Berry Powder

Lyophilized maqui berry (*Aristotelia chilensis*) powder was obtained from Nanuva (Los Ángeles, Chile). The microencapsulation procedure was adapted from [27] (Figure S2). Maltodextrin was dissolved in distilled water at a 1:6 (*w*/*v*) ratio and mixed at 30 °C for 1 h. Separately, maqui berry powder was dispersed in 180 mL of 20% (*v*/*v*) ethanol at 70 °C for 30 min under magnetic stirring. The resulting preparation was filtered through a 0.45 µm membrane. The filtered maqui preparation was subsequently combined with the maltodextrin solution to obtain a formulation corresponding to 6% of the initial maqui berry powder and 94% maltodextrin on a dry-weight basis. The mixture was homogenized using a rotor–stator high-shear homogenizer (T25 ULTRA-TURRAX, IKA-Werke GmbH & Co. KG, Staufen, Germany) at 8000 rpm for six 30 s homogenization cycles, with 30 s resting intervals between cycles. The homogenized feed was spray-dried using a bench-top spray dryer (B-290, Büchi Labortechnik AG, Flawil, Switzerland). The inlet and outlet temperatures were 140 and 100 °C, respectively, using a 0.7 mm nozzle and a feed rate of 30–40 g/min. Five batches were produced. The overall microencapsulation yield was 73%, calculated as the ratio of the mass of microencapsulated powder recovered after spray drying to the total initial mass of maqui berry powder and maltodextrin used in the formulation, expressed as a percentage. The resulting microencapsulated powder was stored in light-protected glass containers at room temperature and used within one week for gummy preparation.

### 4.4. Preparation of Maqui Berry Gummy Formulations

Two maqui berry gummy formulations were prepared: an unencapsulated maqui berry gummy (UMBG) and a microencapsulated maqui berry gummy (MMBG). For both formulations, a hydroalcoholic preparation (230 mL) containing 0.438 g of maqui berry powder and 10.95 g of maltodextrin was used as the active mixture. Each gummy was prepared from 10 mL of this mixture, providing approximately 19 mg of maqui berry and 476 mg of maltodextrin per gummy. Based on the nutritional information provided by the manufacturer for the ingredients, each gummy provided approximately 14.5 kcal, 2.09 g of protein, 0 g of total fat, 1.23 g of available carbohydrates, 0.11 g of total sugars, and 0.04 mg of sodium. In MMBG, maqui berry was present as a freeze-dried microencapsulated powder (6% maqui, 94% maltodextrin), whereas in UMBG the same amount of unencapsulated maqui berry powder was dispersed in the matrix. Additional maltodextrin was added to UMBG so that both formulations had comparable carrier matrices.

The remaining ingredients were sugar-free jelly with berry flavor (Vivo^®^-Carozzi, Santiago, Chile) and a maqui–maltodextrin mixture, either microencapsulated (MMBG) or unencapsulated (UMBG), respectively. For each gummy, 3.6 g of sugar-free berry-flavored jelly was combined with 10 mL of distilled water/ethanol (80:20, *v*/*v*), and all ingredients were weighed, mixed, and heated at 40 °C for 15 min. The mixture was transferred into silicone molds and maintained at 4 °C for 30 min. Each gummy weighed approximately 8 g. For the acute intervention, participants consumed three gummies (24 g in total), providing 57 mg of maqui berry equivalent and 693 mg of quercetin equivalents (QE), according to the Folin–Ciocalteu assay per intervention. The same number and total mass of gummies were administered in the UMBG and MMBG conditions.

### 4.5. Sensory Acceptability Testing

Sensory acceptability of the two maqui berry gummy formulations was evaluated in 66 untrained consumer panelists aged 18–60 years (41 women and 25 men). Panelists were recruited among students, staff, faculty members, and members of the local community at the Universidad de Valparaíso. Fifty-four percent of the panelists were between 18 and 25 years of age. Testing was conducted on a single day in the sensory evaluation facilities of the Food Science Laboratory, Faculty of Pharmacy, Universidad de Valparaíso.

Each panelist evaluated both the unencapsulated maqui berry gummy (UMBG) and the microencapsulated maqui berry gummy (MMBG). Samples were served at room temperature; each participant received one gummy per sample. The two formulations (sample A and sample B) were presented in a blinded fashion in a fixed order (A followed by B), and participants were not informed which formulation they were tasting. Water was provided for palate cleansing between samples, and participants were instructed to rinse their mouth between tastings to minimize potential bias. Participants were given as much time as needed to complete the evaluation.

Acceptability was assessed using a five-point hedonic scale ranging from 1 (“extremely dislike”) to 5 (“extremely like”). Panelists rated appearance, color, bitterness, astringency, sweetness, softness, smoothness, smell/aroma, and taste. All 66 panelists evaluated both formulations, allowing for paired comparisons between UMBG and MMBG. This sensory test was conducted with adult volunteers and involved minimal risk. The study, including the sensory evaluation component, was approved by the Ethics Committee of the Faculty of Pharmacy, Universidad de Valparaíso (CBI 014/2022). All panelists received written and verbal information about the study procedures and provided verbal informed consent before participation.

### 4.6. Study Design and Acute Interventions

This exploratory acute study used a three-condition within-subject design in which each participant completed three separate intervention visits, with a one-week washout between visits. The one-week washout period was selected based on previous crossover studies investigating polyphenol-rich interventions, in which a 7-day washout was used between experimental conditions together with restrictions on polyphenol-rich foods to minimize potential carryover effects [41,43]. The three conditions were (1) Control, consisting of 150 g of white bread providing approximately 75 g of available carbohydrate; (2) UMBG, consisting of 150 g of white bread co-consumed with three unencapsulated maqui berry gummies (24 g total); and (3) MMBG, consisting of 150 g of white bread co-consumed with three microencapsulated maqui berry gummies (24 g total). Each participant therefore served as their own control. Baseline refers to the fasting time-0 measurement obtained immediately before the meal at each visit; Control refers to the white bread only intervention condition.

The order of the three intervention conditions was fixed, and all participants completed the visits in the same sequence: Control first, followed by UMBG and then MMBG. On each study day, participants attended the study center in the morning after an overnight fast of typically 8–10 h (maximum 12 h). All visits for a given participant were scheduled at approximately the same time between 08:00 and 10:00 h to minimize circadian variability. A fasting blood sample was obtained immediately before consumption of the assigned test meal (time 0; baseline for that visit). Participants then consumed the corresponding intervention within 5 min, and postprandial measurements were performed over the subsequent 120 min. Visits were conducted approximately one week apart and under standardized pre-test conditions, including a fixed morning schedule and prespecified dietary and physical activity restrictions. Specifically, participants were instructed to maintain their usual diet and lifestyle throughout the study, to avoid vigorous physical activity, to refrain from alcohol consumption, and not to use antioxidant supplements during the study period. Adherence to these instructions was reinforced between visits and at each study visit.

The white bread only condition served as the no maqui control. A matched placebo gummy was not included; therefore, effects observed after UMBG or MMBG cannot be fully separated from potential effects of the gummy matrix itself. The study focused on objectively measured metabolic and antioxidant response biomarkers and on within-participant comparisons across the three acute conditions. The study protocol was approved by the Ethics Committee of the Faculty of Pharmacy, Universidad de Valparaíso (CBI 014/2022), and all participants provided written informed consent before enrollment. The study was registered at ClinicalTrials.gov as “Gummy Candies With Maqui Berry (MaquiGUM)” (identifier NCT07373795).

### 4.7. Participants

Participants were recruited in Valparaíso Province, Chile, over a one-month period through social media and printed posters. Ten adults aged 18–59 years with overweight or obesity were screened for eligibility and enrolled in the acute intervention study. All participants completed the three experimental conditions; no participants were excluded during screening, and no dropouts occurred.

Eligibility criteria included a body mass index (BMI) ≥ 25.0 kg/m^2^, absence of diagnosed noncommunicable chronic diseases, and sedentary status. Physical activity status was assessed by self-report, including questions regarding the type, duration, frequency, and intensity of physical activity. Intensity was categorized as light, moderate, or vigorous. For the purposes of participant eligibility, a sedentary/physically inactive status was operationally defined as performing less than 30 min of light-intensity physical activity and no moderate- or vigorous-intensity physical activity. Exclusion criteria included the use of glucose-lowering medications, antioxidant-containing supplements, known allergy or intolerance to maqui berry or any component of the gummy formulations, and pregnancy or lactation. Habitual consumption of polyphenol-rich foods was assessed by self-report, and participants were instructed to maintain their usual dietary habits throughout the study. In addition, participants were instructed to avoid polyphenol-containing supplements and to avoid major changes in their usual food intake before each intervention visit. Dietary intake was assessed before each visit using a food consumption questionnaire covering the preceding seven days, allowing dietary patterns and polyphenol intake to be monitored across the experimental sessions. Participants were also instructed to maintain their usual level of physical activity throughout the study and to avoid changes in their habitual exercise routine before each intervention visit.

At enrollment, body weight was measured using a Seca 803 digital scale (Seca GmbH & Co. KG, Hamburg, Germany), and height was measured using a Seca 213 portable stadiometer (Seca GmbH & Co. KG, Hamburg, Germany). BMI was calculated as weight divided by height squared (kg/m^2^). Waist circumference and mid-upper arm circumference were measured using a Seca 201 measuring tape (Seca GmbH & Co. KG, Hamburg, Germany). Waist circumference was measured with the participant standing upright and their arms relaxed at the sides and the tape positioned horizontally at the midpoint between the lowest palpable rib and the iliac crest, at the end of a normal expiration, without compressing the soft tissue. Mid-upper arm circumference was measured at the midpoint between the acromion process and the olecranon, with the arm relaxed and hanging alongside the body. Triceps skinfold thickness was measured at the same midpoint on the posterior aspect of the upper arm using a calibrated Lange metal skinfold caliper (Beta Technology, Inc., Washington, DC, USA), with the skinfold held vertically and parallel to the long axis of the arm. All measurements were performed according to standardized anthropometric procedures [44].

### 4.8. Dietary Assessment

Habitual dietary intake was assessed using a food consumption questionnaire based on the Chilean Food Consumption Survey (Encuesta de Consumo Alimentario en Chile, ENCA) developed under the auspices of the Chilean Ministry of Health. The questionnaire was modified for the present study to quantify the consumption of dietary fiber and selected compounds with antioxidant properties, including vitamin C, vitamin E, carotenoids, and polyphenols. The questionnaire was administered before each of the three intervention visits and assessed food consumption during the preceding seven days. Food items included cereals, vegetables, fruits, legumes, oils, foods rich in unsaturated fats, and chocolate.

Reported food consumption frequencies and portion sizes were converted into estimated daily amounts for each food item expressed in grams per day (g/day). These amounts were matched with the corresponding nutrient and bioactive compound composition data from the 2018 version of the Chilean Food Composition Table [45] and the USDA FoodData Central database [46] to estimate daily intake of dietary fiber, vitamin C, vitamin E, and carotenoids. Total polyphenol intake was estimated using the INTA Database of Antioxidant Activity and Total Polyphenol Content in Foods Produced and/or Consumed in Chile [47]. For each food item, the estimated daily amount consumed was multiplied by the corresponding nutrient or bioactive compound content per 100 g of food and divided by 100. The resulting values were summed across food items to estimate the participants’ daily intake of dietary fiber (g/day), vitamin C (mg/day), vitamin E (mg/day), carotenoids (mg RE/day), and total polyphenols (mg GAE/day).

### 4.9. Blood Sample Collection and Processing

Venous blood samples were collected at baseline (0 min), following an overnight fast of typically 8–10 h, with a maximum of 12 h, and 120 min after consumption of each intervention during each of the three intervention visits. Blood was collected into BD Vacutainer^®^ Hemogard™ (Becton, Dickinson and Company, Franklin Lakes, NJ, USA) tubes containing 75 USP units of sodium heparin per tube. For insulin and glucagon-like peptide-1 (GLP-1) measurements, blood samples were centrifuged within 30 min of collection at 2000× *g* for 10 min at 4 °C. The resulting plasma was aliquoted and stored at −80 °C until analysis. For antioxidant response measurements, blood samples were centrifuged within 30 min of collection at 2000× *g* for 15 min to separate plasma from erythrocytes. Plasma was immediately aliquoted and stored at −80 °C until total reactive antioxidant power (TRAP) analysis. The erythrocyte fraction was washed three times with washing buffer and centrifuged at 3000× *g* for 15 min after each wash. The washed erythrocytes were then aliquoted and hemolyzed with hypotonic solution at dilution ratios of 1:10 for catalase (CAT) and 1:28 for superoxide dismutase (SOD) and stored at −80 °C until analysis.

### 4.10. Antioxidant Response Biomarkers Determination

#### 4.10.1. Total Reactive Antioxidant Power (TRAP)

Plasma total reactive antioxidant power (TRAP) was determined according to [48], with minor modifications. Briefly, 10 μL of plasma was added to a 1:1 mixture containing 150 μM 2,2′-azino-bis(3-ethylbenzothiazoline-6-sulfonic acid) (ABTS) and 10 mM 2,2′-azobis(2-amidinopropane)dihydrochloride (AAPH), which had been preincubated at 45 °C for 30 min. Absorbance was measured at 734 nm 10, 30, and 50 s after sample addition. Antioxidant capacity was quantified using Trolox as the reference standard and expressed as μM Trolox equivalent antioxidant capacity (TEAC).

#### 4.10.2. Total Protein Measurement

Total protein concentration in erythrocyte lysates was determined for normalization of SOD and CAT activities using the Pierce BCA Protein Assay Kit (Thermo Fisher Scientific, Rockford, IL, USA) according to the manufacturer’s instructions. An albumin standard supplied by the manufacturer was used to generate the calibration curve. SOD and CAT activities were subsequently normalized to total protein content and expressed as U/mg protein.

#### 4.10.3. Superoxide Dismutase (SOD)

Superoxide dismutase (SOD) activity was determined using a xanthine/xanthine oxidase–cytochrome *c* assay as previously described [49]. Briefly, 5 μL of erythrocyte lysate was combined with 0.5 mM xanthine and 20 μM cytochrome *c* in phosphate buffer, pH 7.8. The reaction was initiated through the addition of xanthine oxidase in the presence of 0.1 mM EDTA using the same buffer system as described in the original protocol. Absorbance was monitored at 550 nm using a UV-2601 spectrophotometer (Rayleigh, Beijing Beifen-Ruili Analytical Instrument Co., Ltd., Beijing, China). Samples were analyzed in triplicate. SOD activity was normalized to total protein concentration and expressed as units of enzyme activity per milligram of protein (U/mg protein).

#### 4.10.4. Catalase (CAT)

Catalase (CAT) activity was determined by monitoring the decrease in absorbance associated with hydrogen peroxide decomposition according to the previously described method [50]. The reaction mixture contained 100 μL of 0.3 M H_2_O_2_, 2.9 mL of phosphate buffer (50 mM Na_2_HPO_4_ and 50 mM NaH_2_PO_4_, pH 7.8), and 50 μL of erythrocyte lysate. Absorbance was monitored at 240 nm for 90 s using a UV-2601 spectrophotometer (Rayleigh, Beijing Beifen-Ruili Analytical Instrument Co., Ltd., Beijing, China). Samples were analyzed in triplicate. CAT activity was normalized to total protein concentration and expressed as units of enzyme activity per milligram of protein (U/mg protein).

### 4.11. Postprandial Glucose, Insulin, and GLP-1 Assessment

Capillary blood glucose concentrations were measured at baseline (0 min) and at 15, 30, 60, 90, and 120 min after consumption of each intervention using a OneTouch^®^ Ultra^®^ 2 glucose monitoring system (LifeScan, Inc., Malvern, PA, USA). Glucose concentrations were reported in mg/dL. At each time point, capillary blood was obtained through finger prick and analyzed immediately according to the manufacturer’s instructions. Each intervention visit began under fasting conditions following an overnight fast of approximately 8–10 h, with baseline measurements performed at approximately 10:00 a.m.

Total glucose area under the concentration–time curve (AUC) from 0 to 120 min was calculated for each participant and intervention condition using the trapezoidal rule. The calculation was based on the measured glucose concentrations at 0, 15, 30, 60, 90, and 120 min and represents total AUC rather than incremental AUC.

Plasma insulin concentrations were measured at baseline and 120 min using a sandwich enzyme-linked immunosorbent assay (ELISA; Insulin ELISA, IBL International GmbH, Hamburg, Germany; catalog no. RE53171). Absorbance was measured using a calibrated microplate reader at 450 nm, with background subtraction at 620–630 nm, and insulin concentrations were reported in µIU/mL according to the manufacturer’s instructions. Plasma glucagon-like peptide-1 (GLP-1) concentrations were measured at baseline and 120 min using a Human GLP-1 ELISA Kit (BMS2194; Thermo Fisher Scientific/Invitrogen, Vienna, Austria). The assay is a sandwich ELISA for the quantitative detection of human GLP-1 and reports concentrations in pg/mL. Absorbance was measured at 450 nm, with 620 nm used as the reference wavelength. The assay recognizes both natural and recombinant human GLP-1. Plasma was aliquoted and stored at −80 °C until analysis to minimize potential degradation before analysis. All procedures were performed according to the manufacturer’s instructions.

Because insulin and GLP-1 were measured at only two time points, AUC values were not used to characterize their postprandial responses. Instead, concentrations at baseline and 120 min were analyzed separately, and the within-participant change from baseline was calculated as Δ120 min = concentration at 120 min—concentration at baseline for each intervention condition.

### 4.12. Statistical Analysis

Statistical analyses were performed using GraphPad Prism version 9.0 (GraphPad Software, San Diego, CA, USA) and JAMOVI version 2.5.3 (The jamovi Project, Sydney, Australia). This was an exploratory acute within-subject study involving 10 participants. No formal prospective efficacy-based sample size calculation was performed. Total glucose AUC from 0 to 120 min was the prespecified primary outcome in this report; hormonal and antioxidant measures and dietary correlations were interpreted as additional exploratory outcomes. The previous acute blueberry study involving 10 adults is cited as a methodological precedent [28], rather than evidence that the present study was adequately powered. The small sample limits precision and the ability to detect differences, and findings are interpreted as preliminary observations to inform a subsequent adequately powered trial. We applied a Rout test to verify outliers, but we did not identify any unexpected result. In this sense, no data obtained for a different analysis was excluded.

Objective biomarkers, within-participant comparisons, standardized pre-test procedures described in Section 4.6, and one-week intervals between visits were used to reduce measurement-related concerns and variability. These measures do not establish the absence of period or carryover effects. Because all participants received the same fixed sequence, intervention and period effects cannot be estimated independently, and the direction and magnitude of any resulting bias are unknown [51].

Data distributions were assessed using the Shapiro–Wilk test and visual inspection of the data. Continuous variables were presented as mean ± standard deviation (SD) when approximately normally distributed and as the median [interquartile range (IQR)] when non-normally distributed. All statistical tests were two-sided, and a *p* value < 0.05 was considered statistically significant.

Sensory acceptability scores for the UMBG and MMBG formulations were compared using the Wilcoxon signed-rank test because the same 66 untrained consumer panelists evaluated both formulations. The sensory attributes evaluated were Appearance, Color, Bitterness, Astringency, Sweetness, Softness, Smoothness, Smell, and Taste. Sensory data were presented as the median [IQR]. Because nine sensory attributes were assessed, *p* values were adjusted for multiple comparisons using the Holm procedure. Estimated dietary intake variables across the Control, UMBG, and MMBG study periods were compared using repeated-measures ANOVA for parametric variables and the Friedman test for non-parametric variables. Data were presented as mean ± standard deviation (SD).

Postprandial glucose concentrations were analyzed using two-way repeated-measures ANOVA, with intervention condition (Control, UMBG, and MMBG), time (0, 15, 30, 60, 90, and 120 min), and the condition × time interaction included in the model. When a significant main effect or interaction was detected, simple-effect pairwise comparisons of intervention conditions within each time point were performed. Total glucose AUC from 0 to 120 min was compared across the three intervention conditions using a one-way repeated-measures analysis of variance (RM-ANOVA). Holm–Sidak’s multiple-comparisons test was used for post hoc pairwise comparisons when appropriate.

For insulin and GLP-1, concentrations at baseline and 120 min were analyzed separately across the three intervention conditions. Changes from baseline were calculated for each participant as Δ120 min = value at 120 min—baseline value. These changes were compared across the Control, UMBG, and MMBG conditions using RM-ANOVA or Friedman’s test, as appropriate, followed by Holm–Sidak-adjusted pairwise comparisons for RM-ANOVA or multiplicity-adjusted post hoc comparisons following Friedman’s test.

For CAT, SOD, and TRAP, baseline and postprandial values were compared within each intervention condition using the Wilcoxon signed-rank test. To directly evaluate whether antioxidant responses differed among interventions, baseline-adjusted analyses from baseline (Δ = postprandial − baseline) were compared across Control, UMBG, and MMBG. SOD, CAT, and TRAP concentrations at 120 min were analyzed using linear mixed-effect models with intervention (Control, UMBG, and MMBG) as a fixed effect, the corresponding baseline value measured at each intervention visit as covariate, and participant as a random intercept to account for the repeated-measures design. Estimated marginal means were used to compare intervention conditions, and post hoc comparisons were adjusted using the Holm method for multiple comparisons. Statistical significance was set at *p* < 0.05.

Exploratory associations between estimated dietary intake and metabolic or antioxidant response variables were examined using Spearman’s rank correlation coefficients. Given the large number of exploratory correlations, *p* values were reported as unadjusted exploratory values. Correlation analyses were considered hypothesis-generating and not interpreted as evidence of causality.

## 5. Conclusions

This small exploratory within-subject study identified acute postprandial response patterns during co-consumption of maqui berry gummies with white bread in adults with overweight or obesity. Both formulations were generally acceptable from a sensory perspective, but microencapsulation did not demonstrate a consistent acceptability advantage. The reported lower total glucose AUC under MMBG versus Control warrants further investigation and does not demonstrate superiority of microencapsulation over UMBG. Hormonal observations are limited by sampling at baseline and 120 min. The reported antioxidant baseline-adjusted analyses do not establish significant differences among conditions or antioxidant protection, with the unencapsulated formulation exhibiting better TRAP results but a decrement in SOD content. The small sample, fixed sequence, and lack of a matched placebo gummy limit causal attribution. Larger, adequately powered randomized trials with counter-balanced sequences and a matched placebo gummy are needed to assess reproducibility and any advantage of both gummy formulations. Longer studies are required to evaluate sustained effects and health benefits.

## Figures and Tables

**Figure 1:**
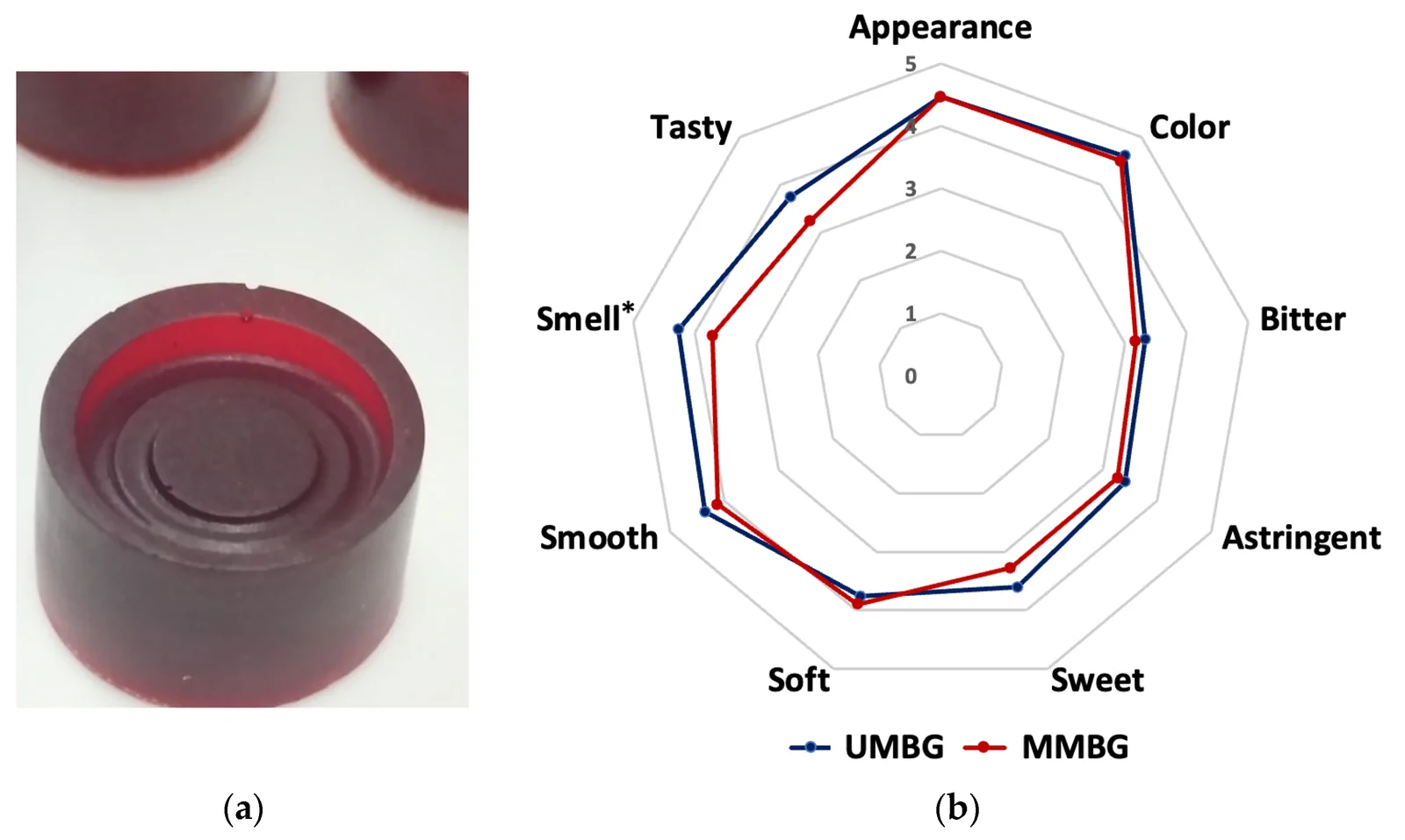
Maqui berry gummy formulation and sensory acceptability. (**a**) Representative image of the gummy candies used in consumer testing. (**b**) Sensory acceptability profile of UMBG and MMBG evaluated using a five-point hedonic scale. UMBG, unencapsulated maqui berry gummy; MMBG, microencapsulated maqui berry gummy. *N* = 66 panelists. * *p* < 0.05.

**Figure 2:**
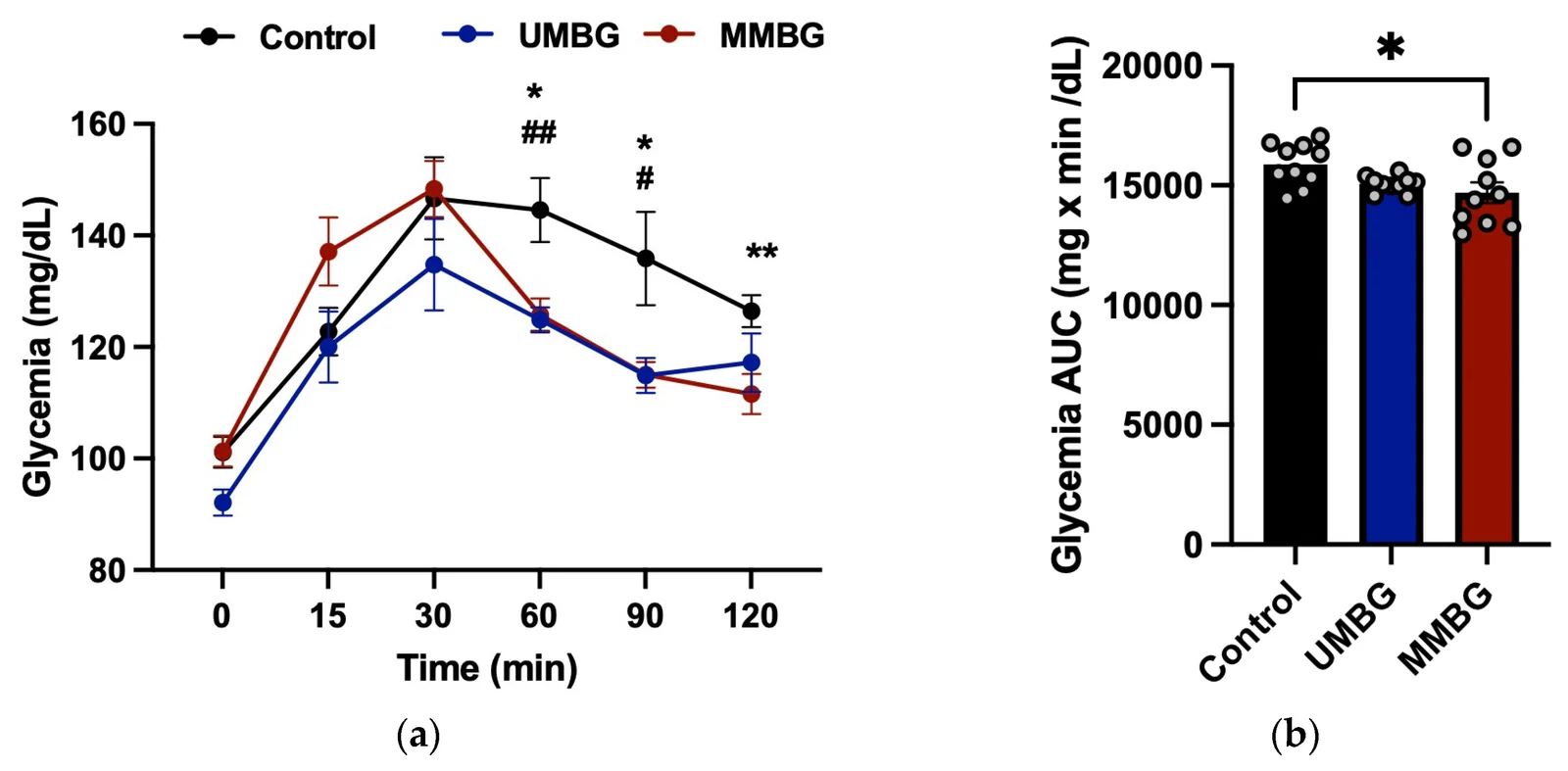
Postprandial glucose response after consumption of gummies. (**a**) Blood glucose concentrations measured at baseline and 15, 30, 60, 90, and 120 min postprandially. Data are presented as mean ± SD, and individual data points are shown where applicable. (**b**) Total glucose area under the curve (AUC; mg·min/dL). Individual values are shown. UMBG, unencapsulated maqui berry gummy; MMBG, microencapsulated maqui berry gummy; AUC, area under curve. * *p* < 0.05 was considered statistically significant (* *p* < 0.05; ** *p* < 0.01, ^#^ *p* < 0.05; ^##^ *p* < 0.01). ^##^: Control vs. UMBG; ^#^ Control vs. MMBG.

**Figure 3:**
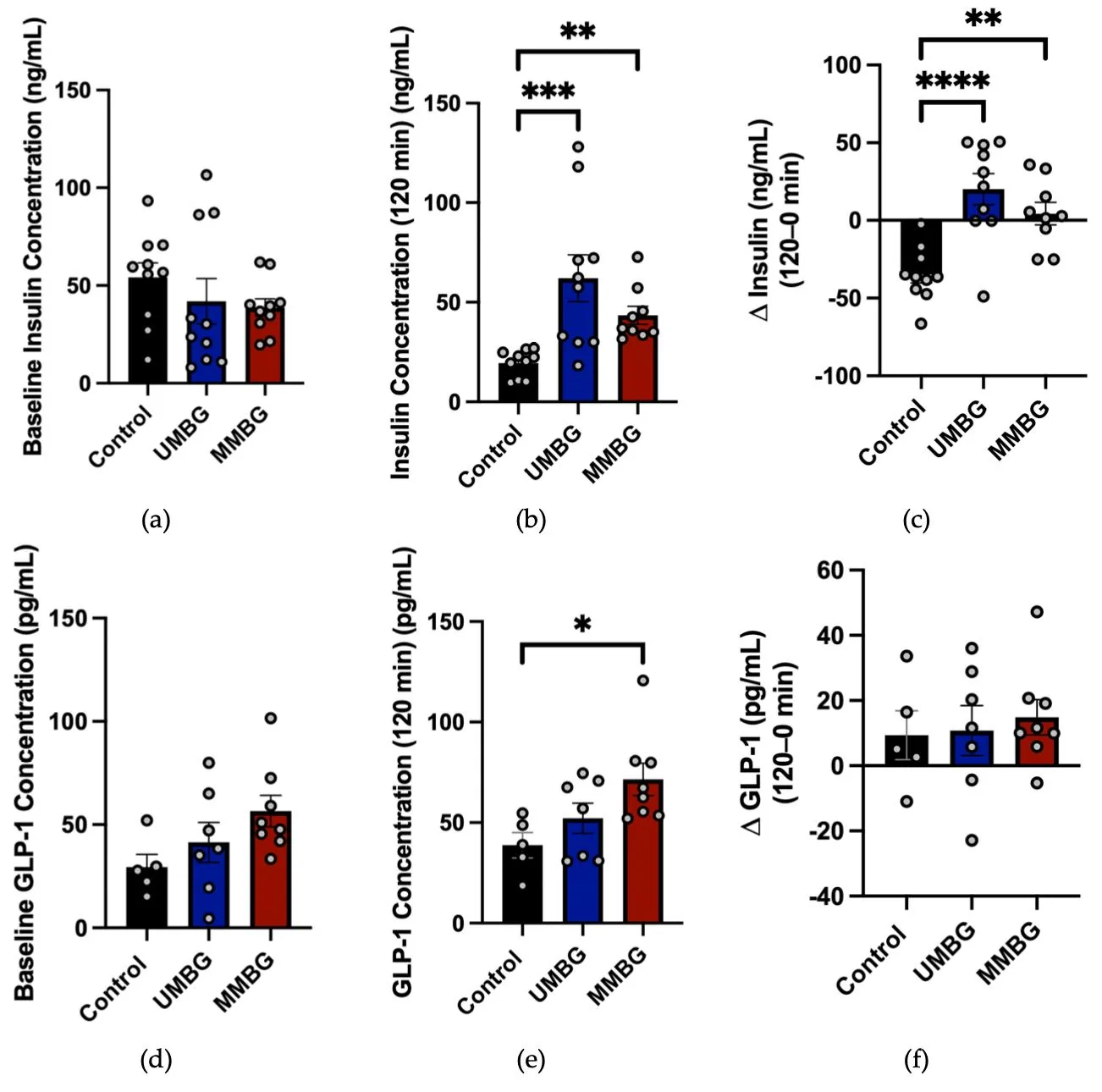
Fasting, postprandial, and change from baseline insulin and GLP-1 concentrations after consumption of each group. (**a**) Fasting insulin concentrations. (**b**) Insulin concentrations at 120 min. (**c**) Change in insulin concentrations from baseline to 120 min (Δ insulin). (**d**) Fasting GLP-1 concentrations. (**e**) GLP-1 concentrations at 120 min. (**f**) Change in GLP-1 concentrations from baseline to 120 min (Δ GLP-1). Individual values are shown. UMBG: unencapsulated maqui berry gummy; MMBG: microencapsulated maqui berry gummy; GLP-1: glucagon-like peptide-1; Δ: 120 min value minus baseline value. * *p* < 0.05 was considered statistically significant (* *p* < 0.05; ** *p* < 0.01; *** *p* < 0.001; **** *p* < 0.0001).

**Table 1:** Sensory acceptability scores of unencapsulated and microencapsulated maqui berry gummies.

Parameters	UMBG	MMBG	*p* Value
Appearance	5.0 [4.0–5.0]	5.0 [4.0–5.0]	1.000
Color	5.0 [4.0–5.0]	5.0 [4.0–5.0]	0.719
Bitterness	3.0 [2.63–4.0]	3.0 [2.0–4.0]	1.000
Astringency	3.0 [3.0–4.0]	3.0 [3.0–4.0]	1.000
Sweetness	4.0 [3.0–4.88]	3.0 [2.25–4.0]	0.158
Softness	4.0 [3.0–5.0]	4.0 [3.0–5.0]	1.000
Smoothness	5.0 [4.0–5.0]	4.0 [3.38–5.0]	0.240
Smell	5.0 [4.0–5.0]	4.0 [3.0–5.0]	0.007 *
Taste	4.0 [3.0–4.88]	3.0 [2.0–4.0]	0.138

Data are presented as median [interquartile range]. Sensory acceptability was evaluated using a five-point hedonic scale ranging from 1 (extremely dislike) to 5 (extremely like). UMBG, unencapsulated maqui berry gummy; MMBG, microencapsulated maqui berry gummy. *N* = 66 panelists. Comparisons between formulations were performed using the Wilcoxon signed-rank test. *p* values were adjusted for multiple comparisons using the Holm method. * *p* < 0.05 after Holm adjustment.

**Table 2:** Antioxidant biomarkers at baseline and postprandially across intervention conditions.

	Control			UMBG			MMBG		
	Baseline	120 min	*p*	Baseline	120 min	*p*	Baseline	120 min	*p*
CAT	1.29	1.29	1.00	1.40	1.30	0.10	1.40	1.30	0.78
(U/mg protein)	[0.97–1.41]	[1.27–1.36]		[1.30–1.50]	[1.30–1.40]		[1.20–1.50]	[1.10–1.50]	
SOD	2.67	2.01	0.05	1.50	1.50	1.00	1.70	1.60	1.00
(U/mg protein)	[2.20–4.49]	[1.90–2.33]		[1.40–1.70]	[1.50–1.60]		[1.60–1.80]	[1.60–1.80]	
TRAP	2.90	2.30	0.03 *	3.20	3.30	0.15	2.00	1.90	0.72
(TEAC μM)	[2.40–3.00]	[2.20–2.50]		[3.10–3.80]	[2.70–3.40]		[1.70–2.20]	[1.60–2.20]	

Data are presented as median [interquartile range]. Baseline to postprandial comparisons within each condition were performed using the Wilcoxon signed-rank test. Control, 150 g of white bread consumed alone; UMBG, unencapsulated maqui berry gummy co-consumed with 150 g of white bread; MMBG, microencapsulated maqui berry gummy co-consumed with 150 g of white bread; CAT, catalase; SOD, superoxide dismutase; TRAP, total reactive antioxidant power; TEAC, Trolox equivalent antioxidant capacity. * *p* < 0.05 was considered statistically significant. *N* = 10 participants.

## Data Availability

The original contributions presented in this study are included in the article/Supplementary Materials. Further inquiries can be directed to the corresponding authors.

## Supplementary Materials

The following supporting information can be downloaded at: https://www.mdpi.com/article/10.3390/ijms27209133/s1.

## Abbreviations

The following abbreviations are used in this manuscript:

GC	Gummy candy
UMBG	Unencapsulated maqui berry gummy
MMBG	Microencapsulated maqui berry gummy
BMI	Body mass index
FFQ	Food Frequency Questionnaires
TRAP	Total Reactive Antioxidant Power
CAT	Catalase
SOD	Superoxide dismutase
AUC	Area under the curve
ROS	Reactive oxygen species
