# Supplementary material for: Acute Postprandial Glycemic and Antioxidant Responses to Maqui Berry Gummies in Adults with Overweight/Obesity: A Small Within-Subject Pilot Study
Source: Int J Mol Sci. 2026 Oct 15;27(20):9133. doi: 10.3390/ijms27209133 (PMC13705431; doi:10.3390/ijms27209133)
Supplement: Supplementary file 1 [file ijms-27-09133-s001.zip › ijms-4567310-supplementary.pdf]

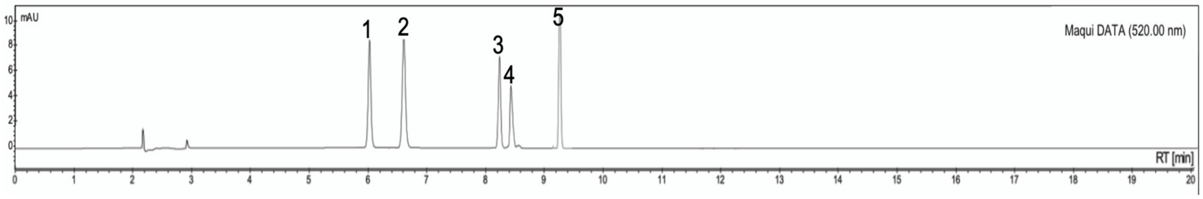

Figure S1. HPLC-UV/Vis chromatogram of the methanolic extract of maqui berry powder recorded at 520 nm. Peak numbers correspond to the tentative assignments reported in Table S1.

Table S1. Tentative anthocyanin profile of maqui berry powder determined by HPLC-UV/Vis.

Anthocyanin	Peak	t_R (min)	λ_{max} (nm)	% area
Delphinidin derivative	1	6.05	277, 345, 523	20.34%
Cyanidin derivative	2	6.65	279, 332, 515	21.58%
Delphinidin derivative	3	8.21	276, 344, 523	17.76%
Cyanidin derivative	4	8.46	278, 334, 517	13.28%
Delphinidin derivative	5	9.30	276, 342, 521	27.04%

t_R: Retention time; λ_{max}: wavelength of maximum absorbance.

Table S2. Estimated background dietary intake across intervention periods.

	Control	UMBG	MMBG	*P value global*
Dietary components				
Dietary fiber (g/day)	42.40 ± 4.13	40.35 ± 4.24	38.88 ± 4.57	0.1088
Vitamin C (mg/day)	254.9 ± 53.84	248.9 ± 58.58	243.4 ± 58.45	0.2231
Carotenoids (mg RE/day)	13.28 ± 2.88	13.12 ± 2.92	12.22 ± 3.01	0.2003
Vitamin E (mg/day)	21.57 ± 4.11	21.22 ± 4.16	19.71 ± 4.15	0.1969
Total Polyphenols (mg GAE/day)	4272.0 ± 766.30	4275.1 ± 775.70	3791.2 ± 648.80	0.1728

Values are presented as mean ± standard deviation. Differences across Control, UMBG, and MMBG were evaluated using repeated-measures analysis of variance for parametric variables, and the Friedman test for non-parametric variables. The global P value is shown in the last column. $P < 0.05$ was considered statistically significant.
UMBG, unencapsulated maqui berry gummy; MMBG, microencapsulated maqui berry gummy; RE, retinol equivalents; GAE, gallic acid equivalents. N = 10 participants.

Table S3. Exploratory Spearman correlations between estimated background dietary intake and antioxidant and metabolic response variables under Microencapsulated- and Unencapsulated-Maqui berry gummy.

	Dietary fiber		Vitamin C		Carotenes		Vitamin E		Polyphenols	
**UMBG**	Spearman coefficient	*P*	Spearman coefficient	*P*	Spearman coefficient	*P*	Spearman coefficient	*P*	Spearman coefficient	*P*
CAT	-0.712	0.07	-0.079	0.84	-0.553	0.12	-0.632	0.07	-0.237	0.54
SOD	0.055	0.89	0.275	0.47	0.165	0.67	0.275	0.47	0.826	**0.01***
TRAP	-0.271	0.48	0.119	0.76	-0.576	0.10	-0.356	0.35	0.153	0.70
Glycemia AUC	0.050	0.91	0.733	**0.03***	-0.067	0.88	-0.400	0.29	0.317	0.41
∆ Insulin	0.021	0.55	0.350	0.31	0.070	0.84	0.020	0.94	0.260	0.47
∆ GLP-1	-0.040	0.96	-0.750	0.06	-0.140	0.78	0.040	0.96	-0.390	0.39
	**Dietary fiber**		**Vitamin C**		**Carotenes**		**Vitamin E**		**Polyphenols**	
**MMBG**	Spearman coefficient	*P*	Spearman coefficient	*P*	Spearman coefficient	*P*	Spearman coefficient	*P*	Spearman coefficient	*P*
CAT	-0.400	0.29	-0.298	0.44	-0.136	0.73	-0.417	0.26	0.145	0.71
SOD	-0.456	0.22	-0.329	0.39	-0.383	0.31	-0.347	0.36	0.639	**0.049***
TRAP	0.126	0.75	0.184	0.64	0.243	0.53	0.276	0.47	-0.586	0.09
Glycemia AUC	0.450	0.23	0.433	0.25	0.583	0.11	0.450	0.23	-0.083	0.84
∆ Insulin	-0.022	0.57	-0.570	0.11	-0.240	0.53	0.180	0.63	0.160	0.68
∆ GLP-1	-0.100	0.84	-0.120	0.79	0.050	0.93	-0.480	0.24	0.330	0.42

UMBG: Unencapsulated-Maqui berry gummy; MMBG: Microencapsulated-Maqui berry gummy; CAT: catalase; SOD: superoxide dismutase; TRAP: total reactive antioxidant power; AUC: Area under the curve; ∆: Delta; GLP-1: Glucagon like peptide. *$P < 0.05$ is significant. N = 10 participants.

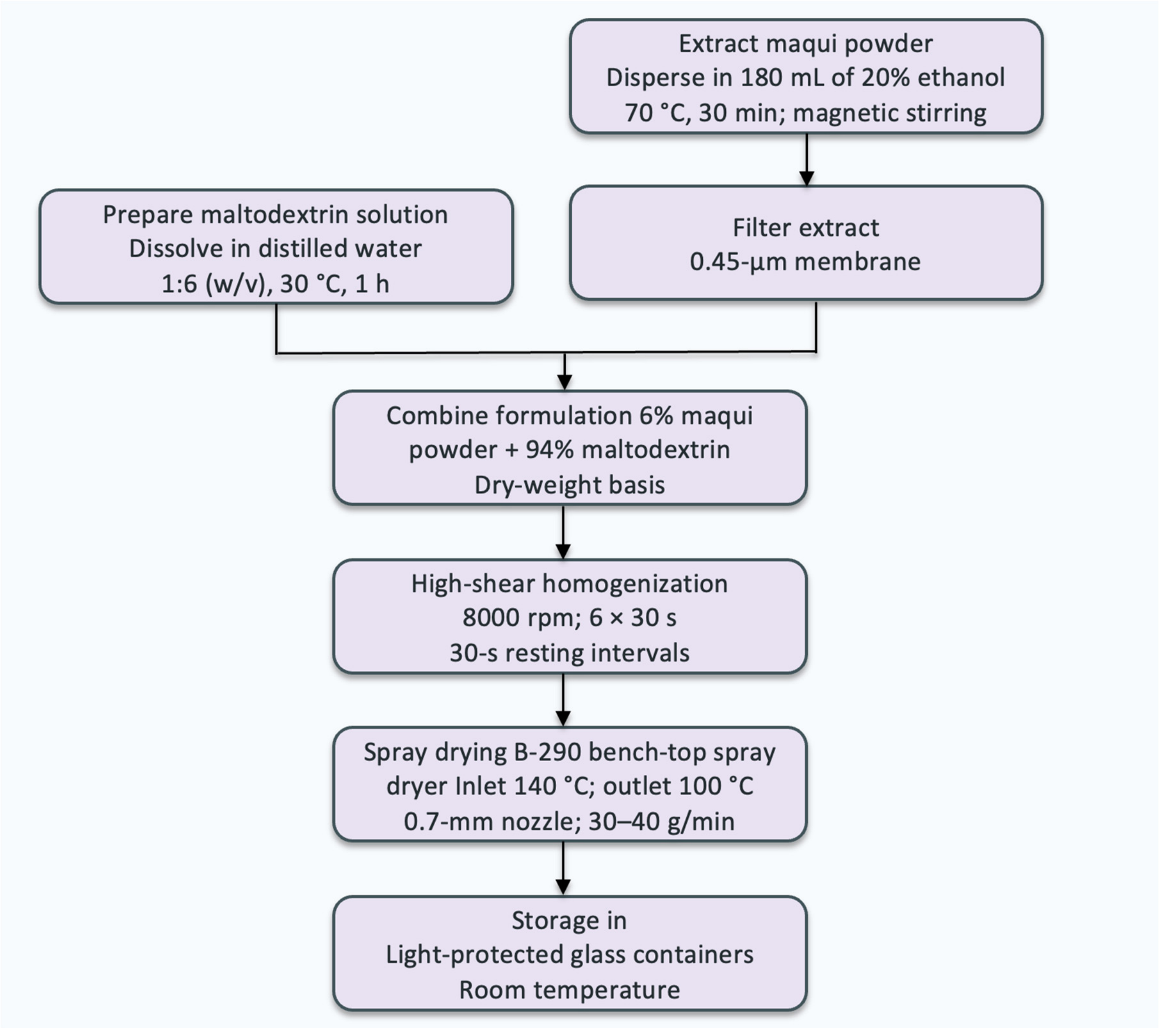

Figure S2. Microencapsulation procedure of Maqui berry powder.
